# MCM8 Is Required for a Pathway of Meiotic Double-Strand Break Repair Independent of DMC1 in *Arabidopsis thaliana*

**DOI:** 10.1371/journal.pgen.1003165

**Published:** 2013-01-03

**Authors:** Wayne Crismani, Virginie Portemer, Nicole Froger, Liudmila Chelysheva, Christine Horlow, Nathalie Vrielynck, Raphaël Mercier

**Affiliations:** 1 INRA, UMR1318, Institut Jean-Pierre Bourgin, RD10, Versailles, France; 2 AgroParisTech, Institut Jean-Pierre Bourgin, RD10, Versailles, France; Karlsruhe Institute of Technology, Germany

## Abstract

Mini-chromosome maintenance (MCM) 2–9 proteins are related helicases. The first six, MCM2–7, are essential for DNA replication in all eukaryotes. In contrast, MCM8 is not always conserved in eukaryotes but is present in *Arabidopsis thaliana*. MCM8 is required for 95% of meiotic crossovers (COs) in *Drosophila* and is essential for meiosis completion in mouse, prompting us to study this gene in *Arabidopsis* meiosis. Three allelic *Atmcm8* mutants showed a limited level of chromosome fragmentation at meiosis. This defect was dependent on programmed meiotic double-strand break (DSB) formation, revealing a role for *At*MCM8 in meiotic DSB repair. In contrast, CO formation was not affected, as shown both genetically and cytologically. The *Atmcm8* DSB repair defect was greatly amplified in the absence of the DMC1 recombinase or in mutants affected in DMC1 dynamics (*sds, asy1*). The *Atmcm8* fragmentation defect was also amplified in plants heterozygous for a mutation in either recombinase, *DMC1* or *RAD51*. Finally, in the context of absence of homologous chromosomes (i.e. haploid), mutation of *AtMCM8* also provoked a low level of chromosome fragmentation. This fragmentation was amplified by the absence of *DMC1* showing that both MCM8 and DMC1 can promote repair on the sister chromatid in *Arabidopsis* haploids. Altogether, this establishes a role for *At*MCM8 in meiotic DSB repair, in parallel to DMC1. We propose that MCM8 is involved with RAD51 in a backup pathway that repairs meiotic DSB without giving CO when the major pathway, which relies on DMC1, fails.

## Author Summary

Species that reproduce sexually have two copies of each chromosome, inherited from their father and mother. During a special cell division called meiosis, these two chromosomes are mixed by homologous recombination to give genetically unique chromosomes that will be transmitted to the next generation. This recombination process is initiated by DNA breaks that must be repaired efficiently to maintain fertility. Using the model plant *Arabidopsis thaliana* we revealed here that the gene *AtMCM8* is required to repair a subset of these DNA breaks. However MCM8 appears to not be required for recombination with the homologous chromosome. Instead MCM8 appears to be involved in a safety system that operates to repair DNA breaks that have not been used for homologous recombination. Interestingly the equivalent gene also has an essential meiotic function in the fly and the mouse. However the three species require *MCM8* for different aspects of meiosis.

## Introduction

Meiosis is a process that occurs in the germlines of sexually reproducing organisms. Two successive rounds of chromosome segregation (meiosis I and II) follow a single round of DNA replication (S phase). The resulting four cells each contain half the genetic content of the pre-meiotic mother cell. The genetic complement of these gametes is a mosaic of the paternal and maternal DNA due to meiotic recombination that occurs between S phase and the first meiotic division [1].

Meiotic recombination begins with programmed DSBs that are dependent on SPO11 and multiple cofactors, including PRD1 in plants [2], [3]. DSBs are subsequently resected to yield 3′ overhangs that invade the homologous chromosome. At this step, two recombinases co-operate to achieve efficient strand exchange with the homolog, RAD51 and DMC1 [4]. RAD51 is a recombinase involved both at mitosis and meiosis while DMC1 is specific to meiosis. Importantly, it has been recently shown in *S. cerevisiae* that only the strand exchange activity of DMC1, and not of RAD51, is required for meiotic crossover formation [5]. RAD51 appears thus to be an accessory factor of DMC1 for meiotic homologous crossover formation, but may also serve as a backup to repair breaks when DMC1 fails [5]. In *Arabidopsis thaliana*, RAD51 is indispensable for repair of meiotic DSBs as shown by the extensive meiotic chromosome fragmentation which occurs at meiosis in *Atrad51* mutants [6], [7]. *At*DMC1 is required for CO formation but not meiotic DSB repair. Indeed, in *Atdmc1* mutant, meiotic DSBs are repaired in a *At*RAD51-dependent manner which does not promote chromosome pairing and does not yield COs between homologs, likely using the sister chromatid as a template [7], [8]. In addition, consistent with a role of RAD51 in helping DMC1 in wild type, the number of DMC1 foci is severely decreased in a *Atrad51* mutant [7], [9], while RAD51 foci are unaffected in *Atdmc1* [9]. Thus two meiotic functions of RAD51 emerge, helping DMC1 to promote COs and promoting DSB repair on the sister without DMC1.

Two other *Arabidopsis* mutants, *sds* and *asy1*, have phenotypes reminiscent of *Atdmc1*, repairing breaks using *At*RAD51 but exhibiting major homologous chromosome pairing defects and making no or few COs [10]–[12]. Both *sds* and *asy1* show localization defects of *At*DMC1 but not of *At*RAD51, suggesting that they work with DMC1 to promote interhomolog recombination [12], [13]. Based on its amino acid sequence, SDS is a cyclin-like protein and ASY1 is a HORMA domain protein making it the likely functional homologue of *S. cerevisiae* Hop1.

DSB repair events form intermediates that are resolved as either crossovers (COs) or non-crossovers (NCOs) (gene conversion). COs are required for accurate segregation of chromosomes during meiosis I and can arise from at least two independent pathways known as class I and class II COs. These two pathways coexist in budding yeast, mammals and *Arabidopsis* [1], [14]–[17]. Class I COs are subject to a phenomenon known as interference, whereby the occurrence of a CO significantly reduces the probability of a CO occurring at an adjacent locus, in a distance dependent manner. This pathway is dependent on the ZMM proteins (defined as ZIP1, ZIP2/SHOC1, ZIP3, ZIP4, MSH4, MSH5, MER3) and, in most eukaryotes, is responsible for the majority of COs during meiosis. Class II COs, that do not display interference, require MUS81 [1], [14]–[17].

Here we addressed the meiotic function of MCM8. MCM8 is a member of the eight MCM family proteins (MCM2–9), that all share a well conserved helicase domain. Together MCM2–7, as a hexamer, form a well characterized DNA helicase, which is essential for replication in all eukaryotes [18]. In contrast, MCM8–9 is not present in all eukaryotes [19], being notably missing in *S. cerevisiae*, *S. pombe* and *C. elegans*, but existing in vertebrates and plants. A study in *Xenopus* showed that MCM8 functions during DNA replication at the elongation stage but it is not required for replication licensing. The *Xenopus* MCM8 protein is the only MCM8 representative for which helicase activity has been demonstrated *in vitro* [20]. MCM8 is also involved in, but not essential for the assembly of the pre-replicative complex in human [21]. Very recently, MCM8 and MCM9 has been shown to be involved in homologous recombination-mediated DNA repair in mouse and chicken somatic cells [22], [23]. MCM8 has also been shown to be involved in meiosis. In the fruit fly (*Drosophila melanogaster*), in which MCM9 has not been identified, *MCM8* (also known as *REC*) is required for 95% of meiotic COs. In contrast to COs, the frequency of NCOs increases in the absence of *Dmrec* [24]. Finally, a very recent study pointed out a role for MCM8, but not MCM9, in meiotic recombination in mouse [22]. Indeed meiocytes in the mouse *mcm8* mutant accumulate DMC1 foci, display synapsis defects and go into apoptosis, consistent with a defect in meiotic DSB repair. The meiotic function of MCM8 has been analyzed only in *Drosophila* and mouse, with contrasting conclusions. This raises the question of the conservation of this function in eukaryotes. The aim of the present study was to further explore the meiotic function of MCM8 by deciphering its role in the model plant *Arabidopsis*.

## Results

### Identification of the *AtMCM8* gene and *Atmcm8* mutations

**Figure 1:**
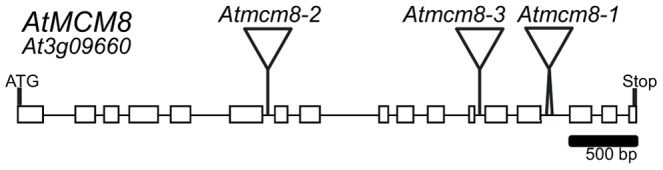
*AtMCM8* gene structure. Exons are represented as black boxes and T-DNA insertions in *Atmcm8-1*, *Atmcm8-2* and *Atmcm8-3* alleles are indicated by triangles.

Phylogenetic analyses of the MCM family [19], [24], showed that the *Arabidopsis* genome contains one clear homolog for each MCM2–9, At3g09660 being the MCM8 homolog. We sequenced the At3g09660 CDS using RT-PCR on mRNA from *Arabidopsis* inflorescences. Because of some differences in splicing sites, the At3g09660 CDS slightly differed from the predicted sequence found in the genebank (NM_111800), measured 2,406 bp and contained 17 exons (Figure 1) (genebank BankIt1577803 MCM8 KC109786). We nonetheless confirmed by reciprocal BLAST analysis and multiple protein alignment that At3g09660 encodes the *Arabidopsis* MCM8 homolog (Figure S1 and [24]).

**Figure 2:**
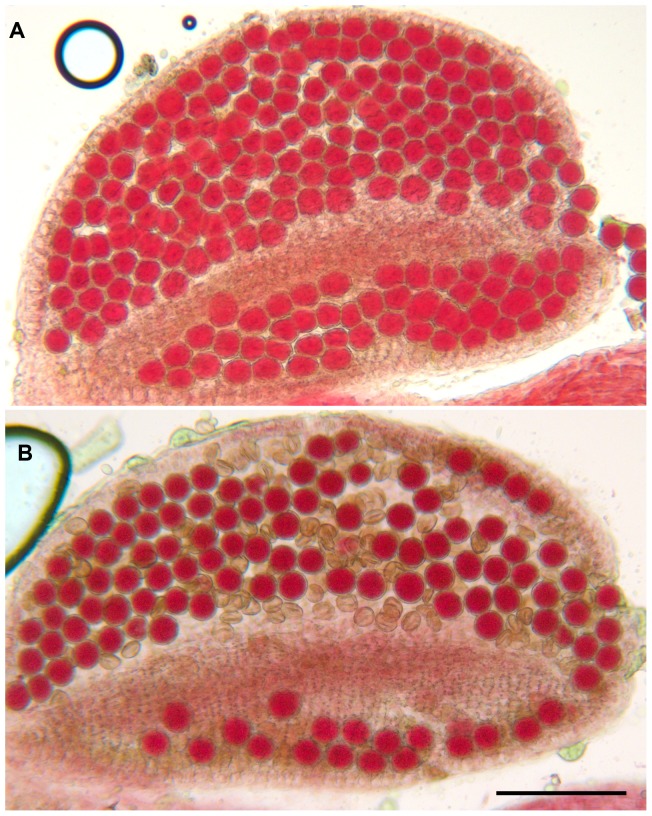
Alexander staining. (A) A wild type anther containing pollen grains that are all viable, as indicated by their red staining and round shape. (B) An *Atmcm8-1* anther containing viable and dead pollen grains as indicated by their abnormal shapes and green coloration. Bar, 100 µm.

We identified three T-DNA insertions from the public collections within the *AtMCM8* gene: *Atmcm8-1*, *Atmcm8-2* and *Atmcm8-3* (Figure 1). Plants homozygous for the insertions showed normal vegetative growth but reduced fertility as shown by Alexander staining of pollen (Figure 2). This phenotype (and others described below) was detected only in homozygotes of each mutant. Moreover seed counts showed that *Atmcm8-1* has significantly less seeds than wild type (44.8±5.2 (n = 41) compared to 52.4±5.8 (n = 77), *Z* test p<10^−13^). Allelism tests showed that the meiotic defects observed (see below) were due to the insertions in *Atmcm8.*

### *Atmcm8* chromosomes fragment during meiosis

**Figure 3:**
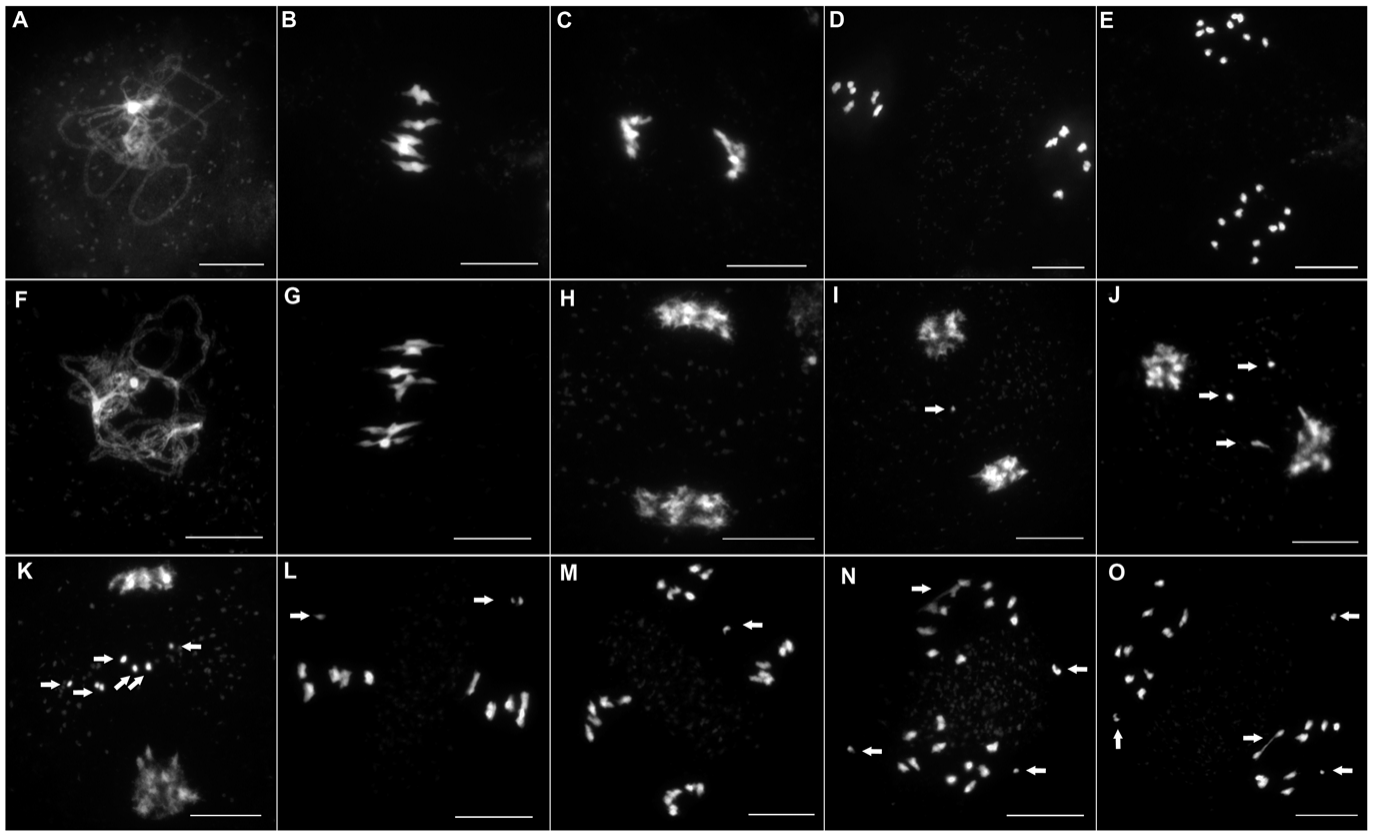
Male meiosis in wild-type and in *Atmcm8*. Male meiosis is shown (A–E) in wild type and (F–O) in *At*mcm8. Chromosome spreads at (A and F) pachytene, (B and G) metaphase I, (C and H–K) end of anaphase I, (D and L) metaphase II, (E and M–O) anaphase II, using DAPI staining. Fragments and chromosome bridges are indicated with arrows. Bar, 10 µm.

**Figure 4:**
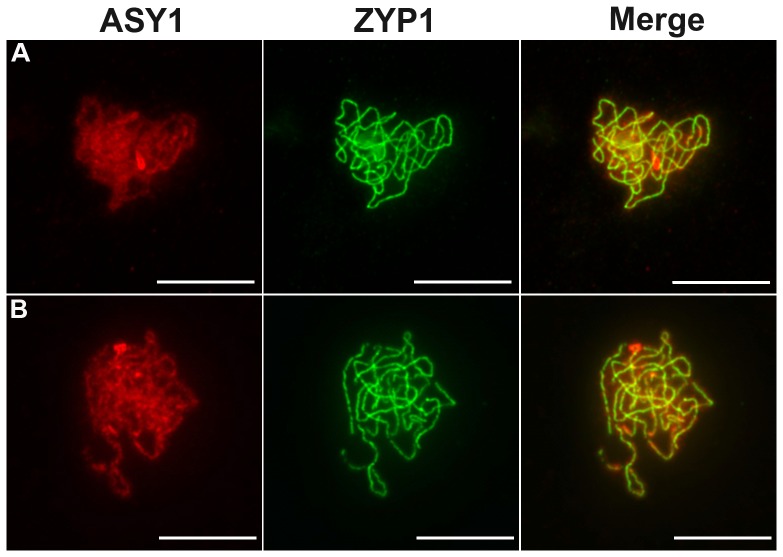
Coimmunolocalization of ASY1 and ZYP1. ASY1 (red), ZYP1 (green) are shown as well as the overlay of both signals (merge) at pachytene in (A) wild type and in (B) *Atmcm8* mutant. In both wild type and mutant the polymerization of the synaptonemal complex, revealed by ZYP1, is completed at pachytene. The ASY1 signal is largely depleted from the chromosomes as the synaptonemal complex forms. Bar, 10 µm.

To investigate if this reduction in fertility was linked to a meiotic defect, we analyzed meiotic progression by DAPI (4′,6-diamidino-2-phenylindole) staining of meiotic chromosome spreads in all three mutant alleles. In wild type meiosis (Figure 3A–3E), chromosomes condense at leptotene. Then, synapsis is initiated at zygotene until its completion in pachytene when the two homologous chromosomes are connected along their entire length by a proteinous structure called the synaptonemal complex [25] (Figure 3A and Figure 4A). Desynapsis occurs at diplotene and further condensation of the chromosomes occurs. Five bivalents continue to condense and become visible at diakinesis. At metaphase I, the five bivalents align on the metaphase I plate (Figure 3B). At anaphase I homologous chromosomes segregate to opposite poles (Figure 3C). At telophase I the two groups of five recombinant chromosomes begin to decondense. At prometaphase II chromosomes recondense and align on the two metaphase II plates (Figure 3D). At anaphase II each of the ten chromosomes segregate their two sister chromatids to opposite poles resulting in four balanced groups of five chromatids (Figure 3E).

**Figure 5:**
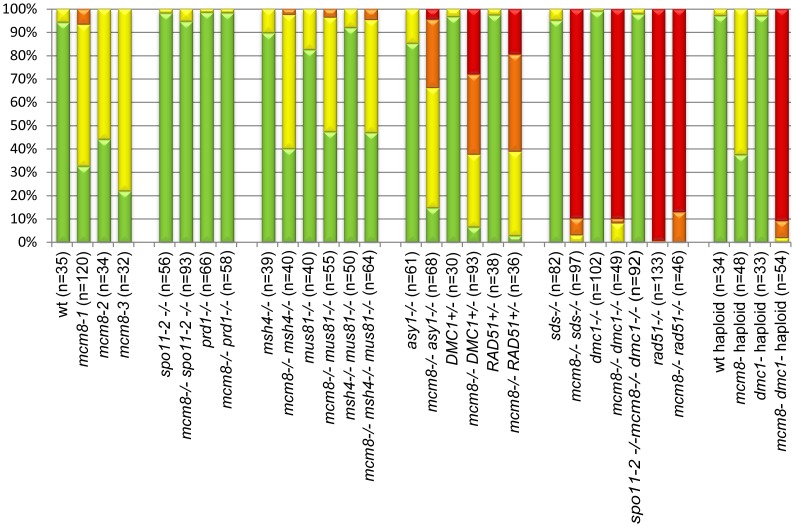
Quantification of chromosome fragmentation levels. For each genotype, a number (indicated in brackets) of late anaphase I/telophase I cells were observed after chromosome spreads and DAPI staining. DAPI-stained bodies observed above the expected number of chromosomes were counted as fragments, and cells were classified has having 0 (green), 1–5 (yellow), 6–10 (orange), or more than 10 fragments (red). The percentage of each class is shown.

In all three *Atmcm8* alleles, meiosis appeared to progress normally from leptotene through to pachytene (Figure 3F) where chromosomes condensed, aligned and fully synapsed like wild type. The completion of synapsis in *Atmcm8* was confirmed by immunolabelling meiotic chromosomes with antibodies against ASY1 and *At*ZYP1 (Figure 4B), that are components of the axial elements and of the transverse filament of the synaptonemal complex, respectively [26], [27]. Chromosomes desynapsed normally during diplotene and we observed five bivalents as condensation progressed during diakinesis, revealing the presence of chiasmata (the cytological manifestation of CO). At metaphase I, five bivalents were systematically observed in all mutant alleles, showing that at least one CO is formed per pair of homologous chromosomes (Figure 3G). Anaphase I proceeded, however chromosome fragmentation was observed in all three *Atmcm8* alleles (Figure 3H–3K), with 1 to 10 chromosome fragments detected in 60 to 80% of the cells (Figure 5). Chromosomes aligned on the metaphase II plate, with fragments dispersed throughout the cell (Figure 3L). Anaphase II proceeded but additional chromosome fragments appeared (Figure 3M–3O). This fragmentation persists at telophase II. We also observed fragmentation in female meiosis showing that *Atmcm8* mutation also affects female meiosis (data not shown).

### Chromosome fragmentation in *Atmcm8* is dependent on meiotic DSB formation

**Figure 6:**
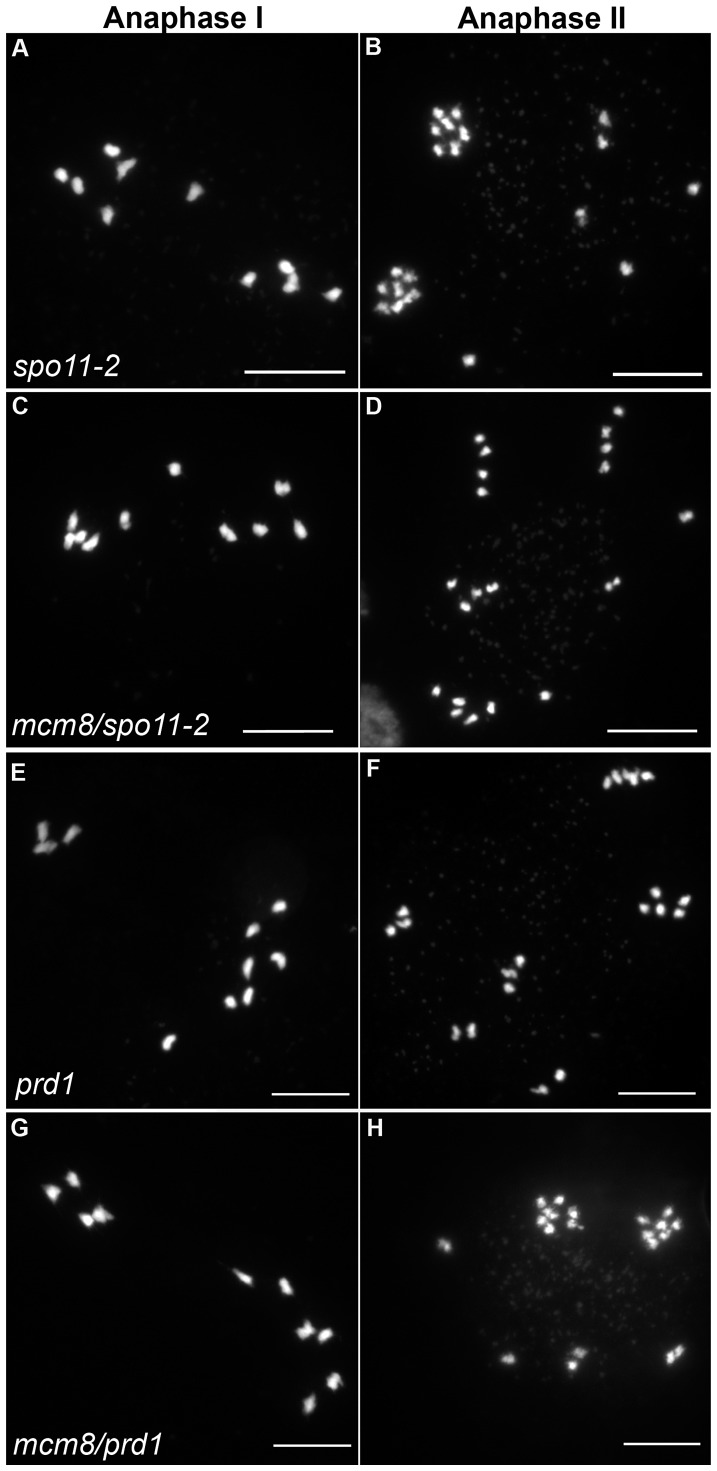
Epistasis tests between *Atmcm8* and two mutants affected in DSB formation. Meiotic spreads with (A–B) *Atspo11-2*, (C–D) *Atmcm8/Atspo11-2*, (E–F) *prd1*, (G–H) *Atmcm8/Atspo11-2* using DAPI staining at anaphase I and anaphase II. Bar, 10 µm.

In *Atspo11-2* and *Atprd1*, no meiotic DSBs are formed and therefore recombination does not occur [3], [28]. Thus at metaphase I, ten univalents are observed and segregate randomly (Figure 6A–6B and 6E–6F). To test whether the chromosome fragmentation seen in *Atmcm8* mutants are dependent on DSB formation or not, we introduced the *Atspo11-2* and *Atprd1* mutations independently into *Atmcm8*. At meiosis, we observed ten univalents at metaphase I in the *Atmcm8/Atspo11-2* or *Atmcm8/Atprd1* and, importantly, the chromosome fragmentation was abolished (Figure 6C–6D and 6G–6H, Figure 5). Therefore, the fragmentation defect of *Atmcm8* is dependent on *At*SPO11-2 and *At*PRD1. Thus, *At*MCM8 is required for efficient repair of the DSBs that initiate meiotic recombination.

### *Atmcm8* does not affect CO frequency

**Figure 7:**
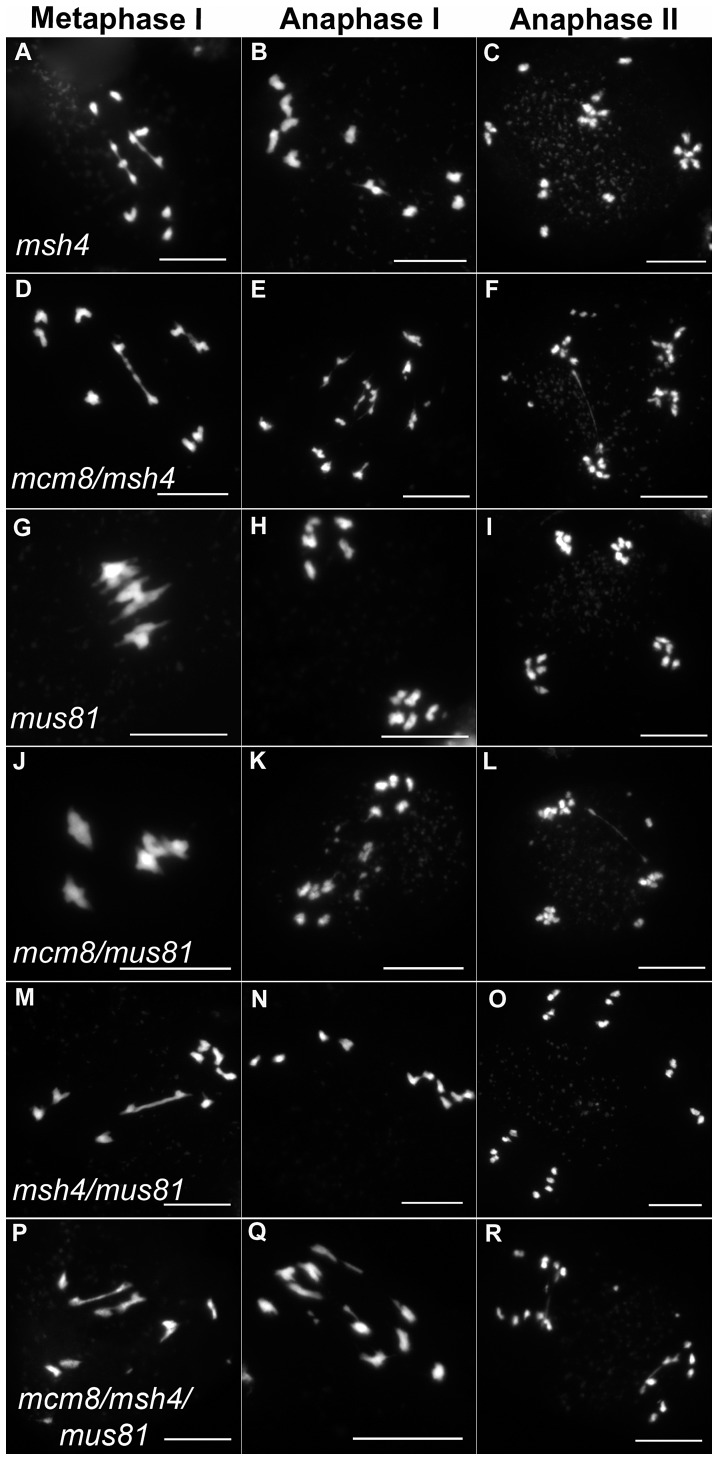
Epistasis tests between *Atmcm8* and mutants affected in crossover formation. Meiotic spreads with (A–C) *Atmsh4*, (D–F) *Atmcm8/Atmsh4*, (G–I) *Atmus81*, (J–L) *Atmcm8/Atmus81*, (M–O) *Atmsh4/Atmus81*, (P–R) *Atmcm8/Atmsh4/Atmus81* using DAPI staining at metaphase I, anaphase I and anaphase II. Bar, 10 µm.

We then tested if the *Atmcm8* fragmentation phenotype is dependent on the presence of any of the known pathways of CO formation, using epistasis tests. We used *Atmsh4* and *Atzip4* that are both required for class I CO formation and *Atmus81* that is required for class II CO formation. In the *Atmcm8/Atmsh4, Atmcm8/Atzip4, Atmcm8/Atmus81* double mutants and the *Atmcm8/Atmsh4*/*Atmus81* triple mutant, we still observed a chromosome fragmentation defect as in the *Atmcm8* single mutant (Figure 5 and Figure 7, data not shown for *Atmcm8/Atzip4*). Thus the *Atmcm8* fragmentation phenotype is independent of MSH4, ZIP4 and MUS81.

In *Atmcm8* and *Atmcm8/Atmus81* we invariably observed five bivalents at metaphase I, suggesting that the formation of class I COs, which account for most of the CO in wild type, is not grossly affected by the *Atmcm8* mutation. This was further supported by counts of *At*MLH1 foci, a marker of class I COs at late prophase of meiosis I [29], [30] (Figure S2), that revealed no significant differences between wild type (10.1±1.4 per cell; n = 81) and the *Atmcm8* mutant (10.3±1.9; n = 86 (*Z* p = 0.55)). In *Atmcm8/Atmsh4* (Figure 6), the residual number of bivalents at metaphase I was unchanged compared to the single *Atmsh4* mutant (1.5±1; n = 91 vs 1.3±1.1; n = 91 (*Z* p = 0.94)), strongly suggesting that class II CO formation is not affected neither by *Atmcm8* mutation. We then measured recombination frequency and crossover interference genetically in *Atmcm8*. This was achieved using tetrad analysis (Fluorescent-Tagged Lines, FTL) which is a visual pollen assay allowing the measurement of multiple COs simultaneously with access to all four chromatids from the same meiosis [31]. Two different sets of adjacent intervals on chromosome 5 have been analyzed, (I5aI5b and I5cI5d), representing four intervals in total. We did not detect any difference in recombination frequency between the *Atmcm8* and wild type for any of these intervals (Table 1, Genetic Distance), consistent with the cytological data. Also, interference, that affects the distribution of crossovers, was unchanged compared to wild type for both sets of adjacent intervals (Table 1, Interference Ratio). Taken together these data suggest that *At*MCM8 is not involved in CO formation. This contrasts from the observation that the absence of MCM8 reduces COs frequency by 95% in *Drosophila* [24].

**Table 1:** Genetic distances and interference in *Atmcm8* using FTLs.

	Genetic distance (cM)			Interference ratio**		
	Wild type	*Atmcm8*	*P* value*	Wild type	*Atmcm8*	*P* value*
I5a	24.2±0.8	22.3±1	0.12	0.27 (chi^2^ p<10^−30^)	0.34 (chi2 p<10^−30^)	0.21
I5b	14.4±0.6	16.1±0.9	0.15			
I5c	5.9±0.4	7.5±0.8	0.09	0.43 (chi^2^ p<10^−5^)	0.30 (chi^2^ p<10^−4^)	0.50
I5d	5.7±0.4	6.3±0.7	0.44			

Values are means ± Standard Error. Number of tetrads: Wild type I5aI5b n = 1986, *Atmcm8* I5aI5b n = 1022, wild type I5cI5d n = 1860, *Atmcm8* I5cI5d n = 646.

* Z-test between wild type and *Atmcm8*.

** The interference ratio is defined as the ratio of genetic distance of I5a with a CO in I5b by the genetic distance of I5a without a CO in I5b. The same was done for the interference ratio between I5c and I5d. Absence of interference would give a ratio of 1 that would tend to 0 with increased interference [57]. The chi square test shows a deviation from 1, and thus the presence of interference [31].

*Mei9/Rad1* is another gene required for the formation of more than 90% of the COs in *Drosophila* [32]. Given the major difference in MCM8 function between *Arabidopsis* and *Drosophila*, we tested the role of *AtRAD1* [33]–[35] in crossover formation in *Arabidopsis*. Cytological analysis showed that the single *Atrad1* mutant has no obvious defect in CO formation. We then analyzed if *At*RAD1 has a minor effect. To achieve this, we constructed a *shoc1/Atrad1* double mutant and a *Atmus81/shoc1/Atrad1* triple mutant to be able to detect a weak reduction in CO formation, in a sensitive context where there are no class I and class II COs. However, this triple mutant was not different from *Atmus81/shoc1* (0.99±0.84 (n = 74) *versus* 1.15±1.28 (n = 75), *Z* p = 0.36) and neither was *shoc1/Atrad1* different from *shoc1* (1.47±1.07 (n = 51) *versus* 1.56±0.86 (n = 32), *Z* p = 0.67). These genes, MCM8 and MEI9/RAD1, are essential for CO formation in *Drosophila* but not in *Arabidopsis* showing divergent functions. However, contrary to RAD1, MCM8 has conserved a meiotic function in *Arabidopsis*.

### The *Atmcm8* DSB repair defect is amplified by *DMC1* mutation

**Figure 8:**
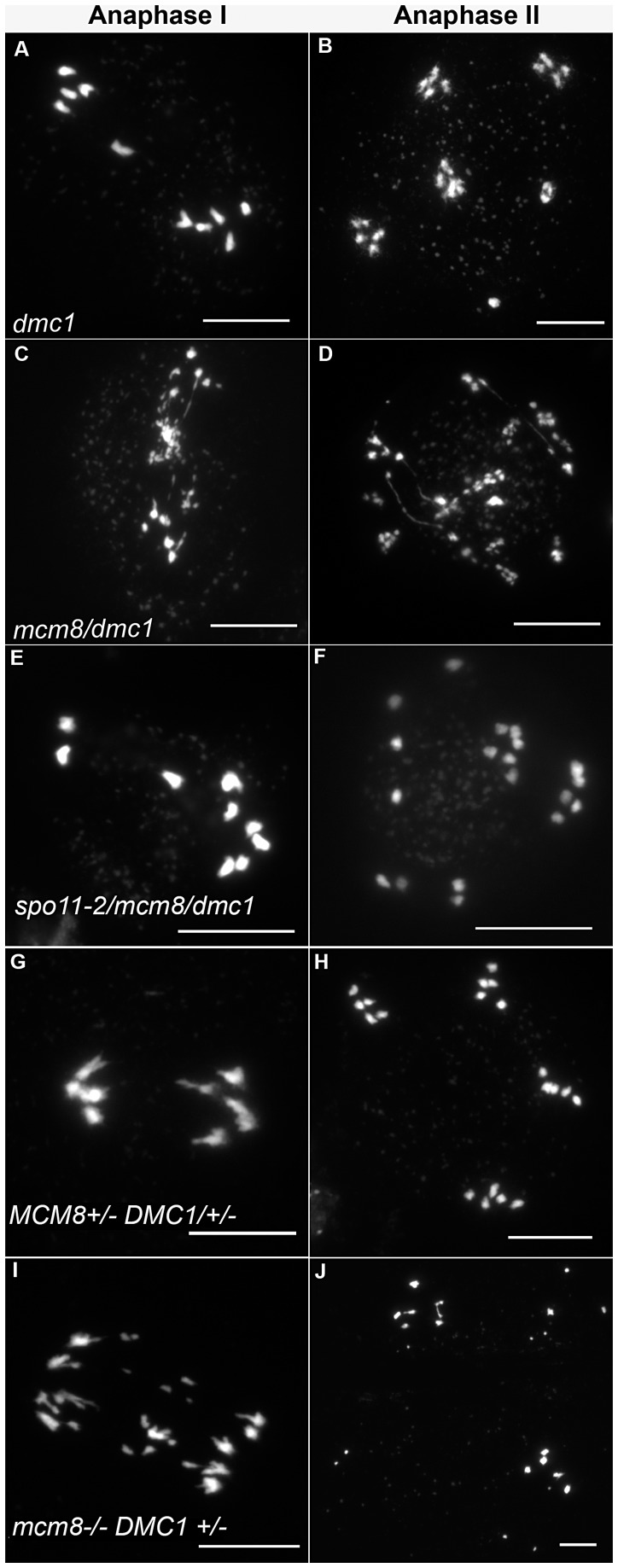
Epistasis tests between *Atmcm8* and At*dmc1*. Meiotic spreads with (A–B) *Atdmc1^−/−^*, (C–D) *Atmcm8^−/−^/Atdmc1^−/−^*, (E–F) *Atmcm8^−/−^/Atdmc1^−/−^/Atspo11^−/−^*, (G–H) *Atmcm8^+/−^/AtDMC1^+/−^*, (I–J) *Atmcm8^−/−^/AtDMC1^+/−^,* using DAPI staining at anaphase I and anaphase II. Bar, 10 µm.

DMC1 is involved at the strand invasion stage of meiotic recombination and *Atdmc1* mutants fail to synapse and to make COs (Figure 8A–8B, 8G–8H). However, DSBs are repaired in *Atdmc1*, in an *At*RAD51-dependent manner, without CO formation, suggesting that the DSBs are repaired on sister chromatids in these mutants [8], [12]. In the *Atmcm8/Atdmc1* double mutant, from metaphase I to the end of the meiosis we observed extensive chromosome fragmentation in all cells, which was much more intense than in the single *Atmcm8* mutant (compare Figure 8C–8D to Figure 3I–3K and see quantification in Figure 5). Consistently, the *Atmcm8/Atdmc1* double mutant was completely sterile whereas *Atmcm8* has moderate fertility reduction and *Atdmc1* produce some residual seeds [8], [12] (Table 2). Mutating SPO11-2 in this *Atmcm8/Atdmc1* double mutant abolished the chromosome fragmentation (Figure 8E–8F, Figure 5), demonstrating that MCM8 and DMC1 act in parallel pathways of meiotic DSB repair.

**Table 2:** Seed per fruit and fragmentation levels in different combinations of double mutants.

Genotype	Seeds per fruit	Bivalent	Fragmentation	Number of DMC1 foci
wt	46.5±1.8 (46)^a^	yes	none	221.6±10.8 (25)
MCM8^+/+^DMC1^+/−^	47.8±2,2 (51)^a^	yes	none	187.3±10.6 (20)
MCM8^+/−^DMC1^+/−^	49.0±1.8 (44)^a^	yes	none	ND
MCM8^+/−^DMC1^+/+^	50.1±1.4 (48)^a^	yes	none	ND
*mcm8* ^−/−^DMC1^+/+^	30.5±2.2 (69)^b^	yes	+	227.8±10.9 (50)
*mcm8* ^−/−^DMC1^+/−^	7.0±1.2 (85)^c^	yes	++	212.9±4.8 (87)
MCM8^+/+^ *dmc1* ^−/−^	1.9±0.2 (76)^de^	no	none	ND
MCM8^+/−^ *dmc1* ^−/−^	1.3±0.2 (65)^de^	no	none	ND
*mcm8* ^−/−^ *dmc1* ^−/−^	0.0±0.0 (50)^e^	no	+++	ND
*mcm8* ^−/−^ *sds* ^−/−^	0.0±0.0 (50)^e^	no	+++	ND
*mcm8* ^−/−^ *asy1* ^−/−^	ND	few	++	ND

Genotype	Seeds per fruit	Bivalent	Fragmentation	Number of DMC1 foci
wt	58.4±1.1 (48)^a^	yes	none	221.6±10.8 (25)
MCM8^+/+^RAD51^+/−^	56.8±1.6 (52)^a^	yes	none	202.7±6.3 (43)
MCM8^+/−^RAD51^+/−^	57.4±1.3 (50)^a^	yes	none	ND
MCM8^+/−^RAD51^+/+^	55.8±1.8 (49)^a^	yes	none	ND
*mcm8^−/−^*RAD51^+/+^	35.4±3.0 (49)^b^	yes	+	ND
*mcm8^−/−^*RAD51^+/−^	10.3±1.6 (87)^c^	yes	++	182.5±8.0 (29)
MCM8^+/+^ *rad51^−/−^*	0.0±0.0 (50)^d^	no	+++	50±11 (21) [7]
MCM8^+/−^ *rad51^−/−^*	0.0±0.0 (50)^d^	no	+++	ND
*mcm8^−/−^rad51^−/−^*	0.0±0.0 (50)^d^	no	+++	51.2±7.0 (8)

Values are means ± Standard Error. The number of fruit or cells counted is indicated in brackets. ND: not determined, a–e: indicates significant differences among groups (Newman Keuls test, p>0.05). Number of crosses indicates fragmentation levels, based on Figure 5.

Furthermore in the *Atmcm8* mutant context, we observed a more drastic meiotic chromosome fragmentation in plants heterozygous for DMC1 (*Atmcm8* ^−/−^ *AtDMC1* ^+/−^) than wild type for DMC1 (*Atmcm8* ^−/−^ *AtDMC1* ^+/+^) (compare Figure 8I–8J to Figure 3I–3K, quantification on Figure 5), accompanied by a strong reduction of fertility (Table 2). However, the fragmentation observed in *Atmcm8* ^−/−^ *AtDMC1* ^+/−^ was less dramatic than in the double mutant (*Atmcm8* ^−/−^ *Atdmc1* ^−/−^) (Figure 5), which is also supported by the fertility levels (Table 2). This is despite the *At*DMC1 mutation being recessive (in an *AtMCM8^+/+^* or *AtMCM8^+/−^* context). Thus, in the absence of *Atmcm8*, the mutation of one of the two copies of DMC1 was enough to enhance fragmentation, which is even more drastic when both *DMC1* alleles are disrupted.

### The *Atmcm8* DSB repair defect is amplified by mutation of *ASY1*, *SDS*, or one copy of *RAD51*

**Figure 9:**
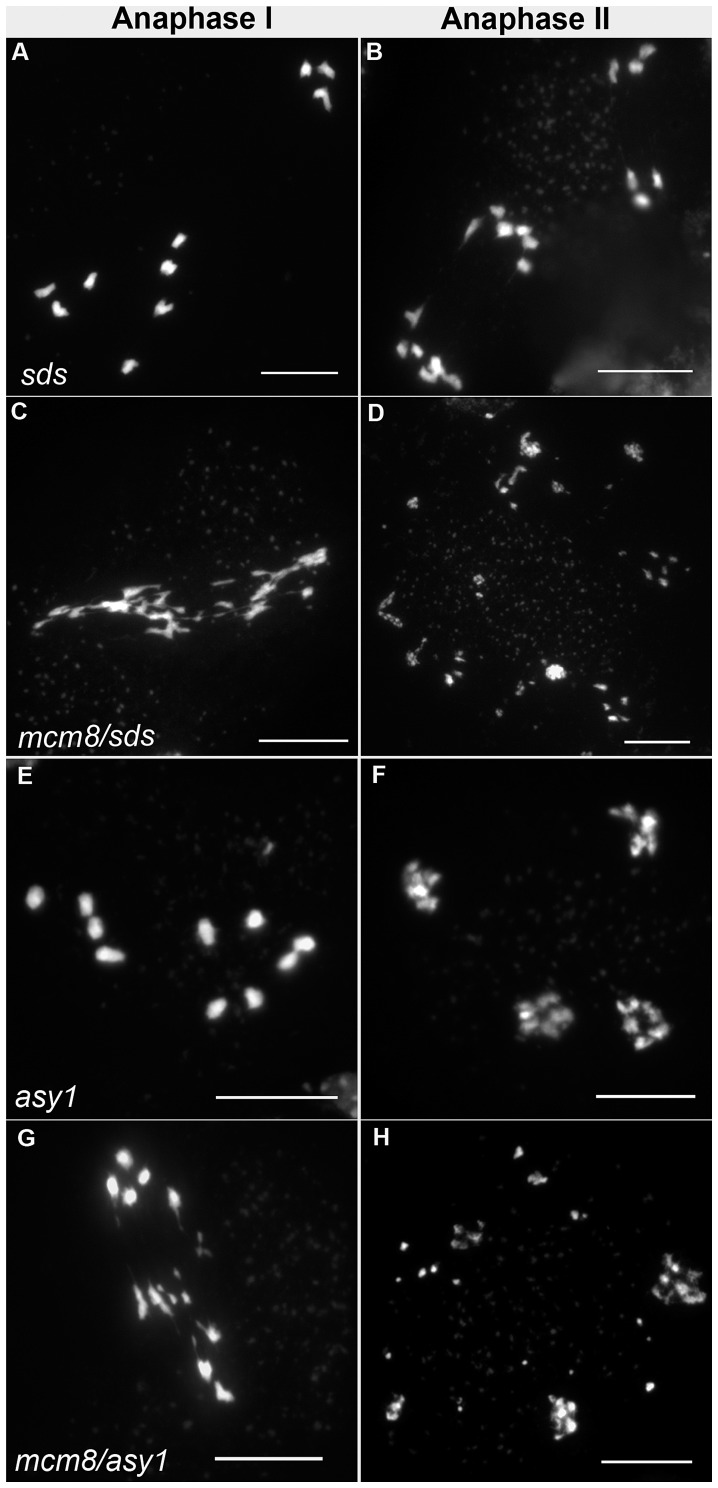
Epistasis tests between *Atmcm8* and *sds* or *asy1*. Meiotic spreads with (A–B) *sds^−/−^*, (C–D) *Atmcm8^−/−^/sds^−/−^,* (E–F) *asy1^−/−^*, (G–H) *Atmcm8^−/−^/asy1^−/^*, using DAPI staining at anaphase I and anaphase II. Bar, 10 µm.

**Figure 10:**
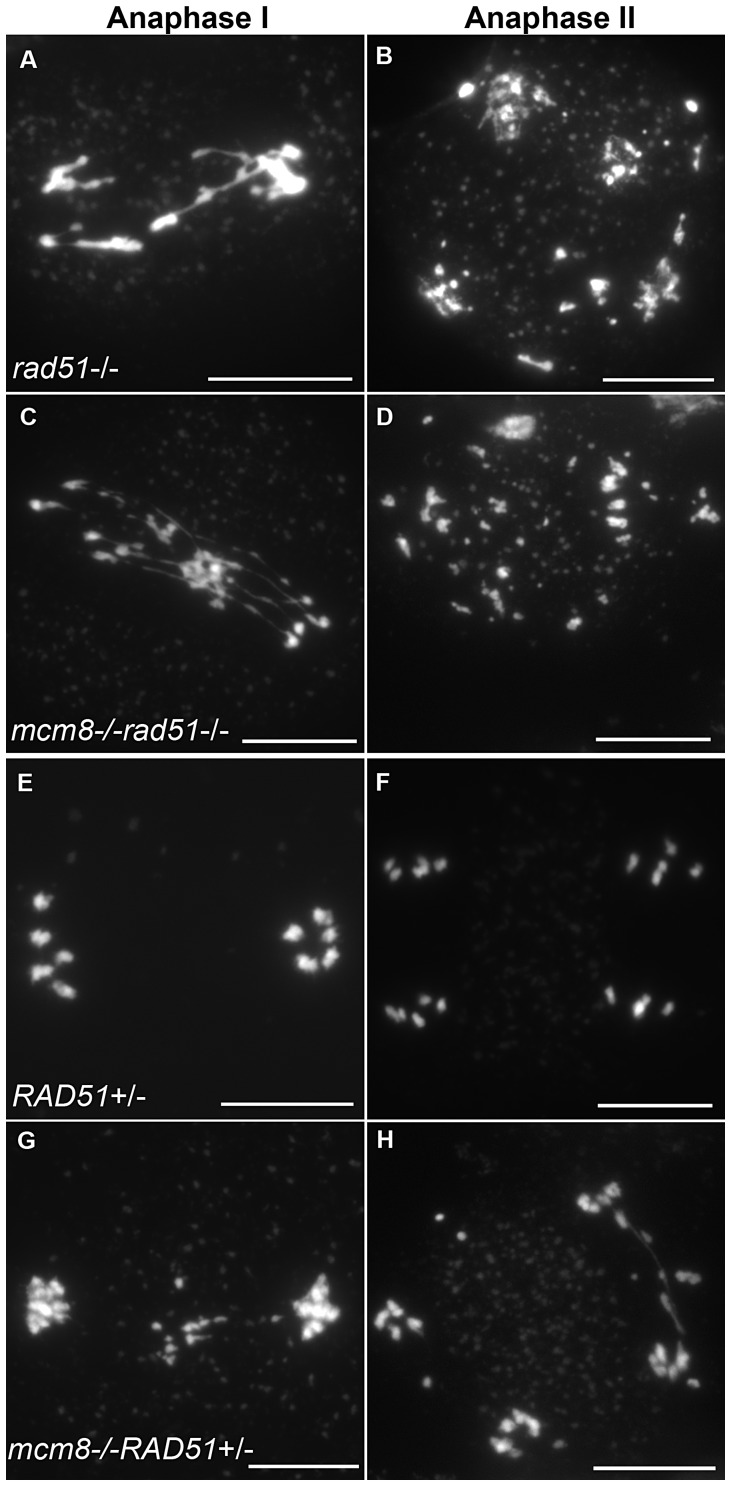
Epistasis tests between *Atmcm8* and *Atrad51*. Meiotic spreads with (A–B) *Atrad51^−/−^*, (C–D) *Atmcm8^−/−^/Atrad51^−/−^*, (E–F) *Atrad51^+/−^*, (G–H) *Atmcm8^−/−^/Atrad51^+/−^*, using DAPI staining at anaphase I and anaphase II. Bar, 10 µm.

Therefore we tested the relationship of *At*MCM8 with ASY1 and SDS, two proteins that are required for normal DMC1 localization [3], [13]. In the *sds* and *asy1* single mutants, COs are greatly reduced (Figure 9E–9F) [10], [11]. In the *Atmcm8/asy1* and *Atmcm8/sds* double mutant, we observed chromosome fragmentation from anaphase I onwards, which was much greater than that seen in the *Atmcm8* ^−/−^ single mutant (compare Figure 9G–9H with Figure 3I–3K, quantification on Figure 5). Thus, mutation of *SDS* or *ASY1* amplified the fragmentation phenotype of *Atmcm8.* Finally, both the single *Atrad51* mutant and the double *Atmcm8/Atrad51* mutant show intense chromosome fragmentation (Figure 10). Interestingly, while *AtRAD51^+/−^* does not show chromosome fragmentation, *Atmcm8^−/−^/AtRAD51^+/−^* showed more chromosome fragmentation that *Atmcm8* (Figure 10, Figure 5). Thus, in the absence of *Atmcm8*, the mutation of one of the two copies of RAD51 was enough to enhance fragmentation.

### *At*DMC1 foci number is unaffected in At*mcm8*

Given the relationship between *DMC1* functional gene copy number and the degree of *Atmcm8*-dependent fragmentation, we looked at DMC1 behavior in *Atmcm8*. No significant difference in DMC1 foci shape or number was observed in *Atmcm8^−/−^* compared to wild type (Table 2). Similarly, we did not detect any differences in number or shape of DMC1 foci in *Atmcm8^−/−^AtDMC1^+/−^* or *Atmcm8^−/−^AtRAD51^+/−^* compared to either wild type or *Atmcm8^−/−^* (Figure S3, Table 2). In the *Atmcm8 Atrad51* double mutant, we observed a marked decrease of DMC1 foci number, which was however similar to what was previously observed in a single *Atrad51* mutant [7] (Table 2). It is intriguing that *Atmcm8^−/−^AtDMC1^+/−^* and *Atmcm8^−/−^AtRAD51^+/−^* exhibit a more drastic meiotic defect than *Atmcm8^−/−^AtDMC1^+/+^*, while DMC1 foci number and shape appear similar. However, it is possible that immunolocalization fails to detect subtle differences in DMC1 protein quantity or dynamics.

### In the absence of homologous chromosomes, DSBs fail to be repaired in the absence of both MCM8 and DMC1

**Figure 11:**
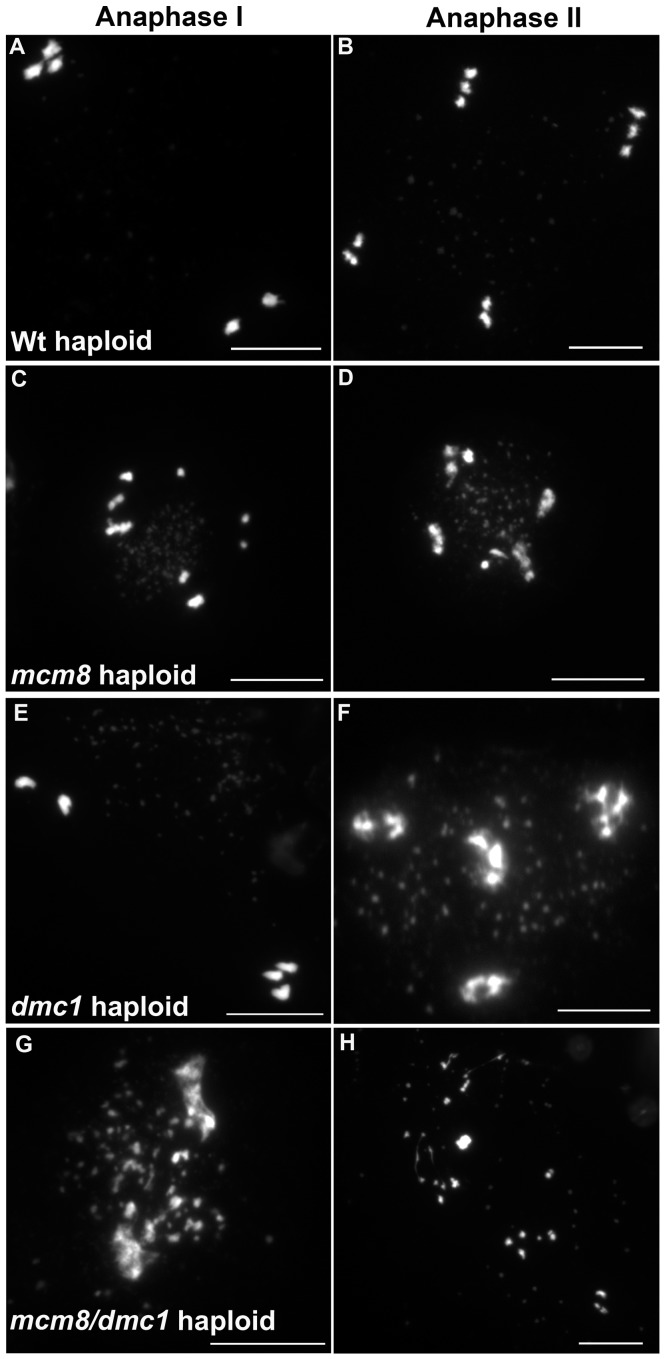
*Atmcm8* haploids during anaphase I and anaphase II. Meiotic spreads with (A–B) wild type *haploid*, (C–D) *Atmcm8 haploid*, (E–F) *Atdmc1 haploid*, (G–H) *Atmcm8/Atdmc1 haploid* using DAPI staining. Bar, 10 µm.

Next we explored the functional relationship between MCM8 and DMC1, in haploid plants, where homologous chromosomes are not present. Thus, the only template available for meiotic DSB repair is the sister chromatid. Meiotic chromosome spreads, in a wild-type haploid, showed that the five chromosomes were intact and segregated randomly at anaphase I [36] (Figure 11A–11B), suggesting that DSBs are efficiently repaired. The haploid *Atmcm8* mutant had a limited fragmentation defect (Figure 11C–11D), similar to the defect in the diploid *Atmcm8* mutant (Figure 5 for quantification). The *Atdmc1* haploid had no fragmentation (Figure 11C–11F). In clear contrast, in the double *Atmcm8/Atdmc1* haploid, we observed extensive meiotic chromosome fragmentation (Figure 11G–11H, see Figure 5 for quantification). This shows that in a haploid context, DSB repair is efficient in wild type and *Atdmc1,* only slightly affected in *Atmcm8*, but ineffective in the *Atmcm8/Atdmc1* double mutant. This suggests that in the absence of a homologous template, *At*MCM8 and *At*DMC1 catalyze DSB repair on the sister chromatid in a redundant manner.

## Discussion

Here *At*MCM8 was shown to be involved in meiotic DSB repair but not CO formation. This study thus revealed a pathway for DNA DSB repair that does not yield COs. This pathway depends on *At*MCM8 and acts in parallel to the *At*DMC1 pathway from which COs originate.

### *At*MCM8 is required for efficient meiotic DSB repair but not for CO formation

*Arabidopsis* MCM8 is required for effective meiotic DSB repair as all *Atmcm8* mutant alleles had a clear, albeit limited, chromosome fragmentation defect at meiosis. The fragmentation is dependent on meiotic DSB formation as it disappears when *AtSPO11-2* or *AtPRD1* is absent. However, in contrast to *Drosophila rec* (*mcm8*) mutants, genetic and cytological data strongly support that CO formation is not affected by *AtMCM8* mutation: (1) In the absence of *AtMSH4* or *At*ZIP4 (class I COs) or *At*MUS81 (class II COs) fragmentation still occurred and the number of bivalents was unchanged. (2) MLH1 foci numbers, a marker of class I COs, were unchanged in *Atmcm8*. (3) The genetic analysis using FTLs revealed no difference in terms of genetic distance and the strength of interference. These data showed that *At*MCM8 acts in a pathway which repairs a subset of meiotic DSB and does not lead to CO formation.

### Two pathways for DSB repair: one dependent on *At*MCM8 and one on *At*DMC1

A striking finding was that *AtMCM8* becomes crucial when the DMC1 pathway was affected. Indeed, we observed a drastic amplification of the *Atmcm8* mutant chromosome fragmentation defect when one of the two allelic copies of DMC1 was mutated, which was even more drastic when both DMC1 copies were mutated. This extensive fragmentation defect reflects a failure of DSB repair, as it is abolished by SPO11-2 mutation. Further, this extensive fragmentation was consistently confirmed in the absence of *AtMCM8* and *SDS*, or *AtMCM8* and *ASY1*. SDS and ASY1 are essential for *At*DMC1 loading/stability [12], [37]. Extensive fragmentation was also observed when one copy of RAD51 was mutated in the *Atmcm8* mutant. A function of RAD51 as a cofactor of DMC1 has been recently identified in yeast [5], and consistently DMC1 foci number is drastically reduced in the *Arabidopsis rad51* mutant [7], [9]. We thus propose that two pathways of DSB repair coexist, one dependent on *At*MCM8 and the other one on *At*DMC1. In the absence of *At*DMC1, efficient DSB repair occurs without CO formation. This repair depends on *At*RAD51 [7], [8], [12] and on *At*MCM8 (this study). Such RAD51-mediated, DMC1-independent, repair also exists in *S. cerevisiae* but is normally inhibited by RAD51 regulators [38]–[42]. Consequently, we suggest that, in the *Atdmc1* context, *At*MCM8 and *At*RAD51 can co-operate to repair DSBs using the sister as a template. In addition to this function, *At*RAD51 is required for the *At*DMC1-dependent pathway (possibly as an accessory factor for the DMC1 strand-exchange activity as shown in yeast [5]) as repair is completely defective in the single *Atrad51* mutant [6], like in the double *Atmcm8/Atdmc1* mutant.

The fact that the fragmentation defect is limited in the single *Atmcm8* mutant, suggests that the *At*MCM8/*At*RAD51 pathway would be essential for a limited number of events in wild type, when DMC1 fails. The repair events promoted by *At*MCM8 are likely not intended to become a CO, as CO formation was not affected in *Atmcm8*, leaving sister chromatid repair or NCOs as the only other known possibilities. The absence of synapsis in *Atdmc1* [7], [8], in which the *At*MCM8/*At*RAD51 pathway must be active, favors the hypothesis of sister chromatid repair. In contrast, the DMC1 pathway promotes CO formation. However, DMC1 foci in wild type, outnumber COs by approximately 25 to 1 [7], [43]. This suggests that repair of many DSBs catalyzed by DMC1 do not become CO, but NCO (that involve the homologous chromosome) or sister chromatid exchange (SCE). In *Arabidopsis,* the genome-wide frequency of NCOs and SCEs is currently unknown. We favor the hypothesis that DMC1 promotes NCOs, as DMC1 promotes synapsis. However, it should be noted that DMC1 is also able to promote SCE, notably in the haploid *mcm8* context. Indeed, only the simultaneous mutation of *AtDMC1* and *At*MCM8 in haploids led to extensive chromosome fragmentation. The capacity of DMC1 to promote inter-sister repair was previously shown in other mutant background in both *Arabidopsis* [9] and yeast [44].

In summary we suggest that two pathways of DSB repair exist in wild type meiosis: The first pathway relies on the strand exchange activity of DMC1, and is also promoted by ASY1, SDS and RAD51 as a co-factor of DMC1 [5]. This pathway generates the COs, but also NCOs and SCEs in a ratio that remains to be determined. The second pathway of the model, which may be viewed as a backup pathway in case of failure of DMC1, relies on the strand exchange activity of RAD51 and the helicase activity of MCM8, and uses the sister chromatid as a template.

### MCM8 function varies among eukaryotes

The function of MCM8 appears to differ markedly in *Arabidopsis* and in *Drosophila*. Interestingly, DMC1 and MCM8 appear to be partially redundant in *Arabidopsis* while the *Drosophila* genome seems devoid of a DMC1 homolog [45]. Thus CO formation in *Drosophila* appears to rely on a RAD51/MCM8 pathway, which has only a minor role in wild type meiotic DSB repair in *Arabidopsis*. The CO pathways appear to differ considerably in the two species, mainly using ZMMs in *Arabidopsis* but not RAD1, and the reverse in *Drosophila,* i.e. RAD1 but not ZMMs (that are absent from the *Drosophila* genome). *Drosophila* appears to be unique, as in distant species like *S. cerevisiae*, mammals and *C. elegans* CO formation depends mainly on ZMM. Adding to the complexity, MCM8 exists in mammals but not in *S. cerevisiae* and *C. elegans* [19], [24]. In mouse, *MCM8* mutation leads to a meiotic arrest, with defects in homologous synapsis and over-accumulation of DMC1 foci before apoptosis, suggestive of defects in DSB repair [22]. We would like to suggest that these defects may compatible with MCM8 being required for a backup pathway in the case of failure of DMC1 to repair breaks, like in *Arabidopsis*. The lack of the backup pathway may lead to the accumulation of DMC1 foci, and a failure to repair a subset of breaks, triggering apoptosis (it is noteworthy that DSB repair defects do not trigger meiotic arrest or apoptosis in *Arabidopsis*). This illustrates the variety of mechanisms that arose in the course of evolution to fulfill the conserved outcome of meiotic DSB repair and CO formation.

In conclusion, our data reveals the meiotic function of MCM8 in *Arabidopsis*. Cytological and genetic analyses showed that *At*MCM8 is involved in DSB repair but it is not a determinant for CO formation. This study identified a new pathway of meiotic DSB repair independent of *At*DMC1.

## Materials and Methods

### Plant material

*A. thaliana* accession Columbia (Col-0) was the wild type reference. *Atmcm8-1* (*Salk_032764*, N532764), *Atmcm8-2* (*Salk_104007,* N604007) *Atmcm8-3* (*Salk_099327*, N599327) were obtained from the collection of T-DNA mutants at the Salk Institute Genomic Analysis Laboratory (SIGnAL, http://signal.salk.edu/cgi-bin/tdnaexpress) [46] *via* NASC (http://nasc.nott.ac.uk/). Other mutants used in this study were *Atspo11-2* (*Gabi_749C12*, N359272) [47], *Atprd1* (*Salk_024703*, N524703) [3], *Atdmc1-3* (*Sail_170_F08,* N871769) [48], *At*rad51 (*Atrad51-1*) [6], *asy1-4* (*Salk_046272,* N546272), *sds-2* (Sail_129_F09, N806294) [12], *Atzip4-2* (*Salk_068052*, N568052) [43], *Atmsh4* (*Salk_136296*, N636296) [49], *mus81-2* (*Salk*_*107515,* N607515), *mus81-3* (*Salk_002761,* N502761) [50], [51], and *shoc1-1* (*Salk_057589,* N557589). *rad1-1* (*uvh1-1*) has a EMS (ethyl methanesulfonate) mutation [33], [34] and was provided by C. White.

### Growth conditions

Plants were cultivated in greenhouse or growth chamber with a 16 h/day and 8 h/night photoperiod, at 20°C and 70% humidity.

### Genetic analysis

Allelism tests were performed by crossing *Atmcm8-1* ^+/−^ with *Atmcm8-2* ^+/−^ and selecting F1 plants hemizygous for both alleles and likewise for *Atmcm8-2* ^+/−^ with *Atmcm8-3* ^+/−^. Double mutants were obtained by crossing heterozygous plants for each mutation and selfing the double heterozygous F1 plants. *Atmcm8*/*Atmhs4*/*Atmus81* triple mutant was identified by crossing *Atmcm8*/*Atmsh4* double heterozygous with *Atmus81* single mutant. As *Atmsh4* and *Atmus81* are linked, a plant heterozygous for *Atmcm8*/*Atmsh4* was self-fertilized and homozygous for *Atmus81* to identify the triple mutant in the offspring. Haploid *Atmcm8* and *Atmcm8/Atdmc1* were obtained by crossing a heterozygous plant for *Atmcm8* or *Atmcm8/Atdmc1* mutations as male and the GEM line as female [36], [52]. In F1, haploid plants of the desired genotype were selected.

### Oligonucleotides for PCR genotyping

Plants of interest were selected by PCR genotyping using diagnostic primer sets. The three *AtMCM8* insertions were genotyped by PCR using following primer combinations to amplify genomic DNA flanking the T-DNA insertions. *Atmcm8-1*: left borders (LB) with LBsalk2 (5′-GCTTTCTTCCCTTCCTTTCTC-3′)/N532764L (5′-AGCGCCATTAGCAAAATGTC-3′) or with LBsalk2/N532764U (5′-GCAGCTTCATTCTGCAAGTG-3′). Wild type allele with N532764U/N532764L. *Atmcm8-2* LB with LBsalk2/N604007L (5′- TCACTACAGCAACGGTGAGC -3′), right border (RB) with RBsalk1 (5′-TCA GAG CAG CCG ATT GTC-3′)/N604007U (5′-GCTGATGGAAGACCTTGTGG-3′). Wild type allele with N604007U/N604007L. *Atmcm8-3* LB with LBsalk2/N599327L (5′-TGGTGTGGAATCAGCAGATG-3′) or with Lbsalk2/N599327U (5′-TGTGTCTCTGTTGCAAAGGC-3′). Wild type allele with N599327U/N599327L. T-DNA right and left borders were analyzed by sequencing PCR products. *At*SPO11-2 wild type allele was amplified using primers 749C12U (5′-GAGCGAGAATTTTTGGTTGG-3′) and 749C12L (5′- CCACAAGGTCAATTCTTCAAC-3′) and mutant allele using N524703L and LBgabi1 (5′-CCCATTTGGACGTGAATGTAGACAC-3′). *At*PRD1 wild type allele was amplified using primers N524703U (5′-AAGTCTGCCCATGGTCACGATTCTCTCTG-3′) and N524703L (5′-GCCTGCTCAAAGGGTCCAGC-3′) and mutant allele using N524703L and LbSalk2. *At*DMC1 wild type allele was amplified using primers N871769U (5′- TTTTTAATTGTTTACAGAGGAAATCAG-3′) and N871769L (5′-TCCACTCGGAATAAAGCAATG-3′) and mutant allele using N871769L and Lb3sail (5′-TAGCATCTGAATTTCATAACCAATCTCGATACAC-3′). *At*RAD51 wild type allele was amplified using primers RAD51-1U (5′-ATGCCAAGGTTGACAAGATTG-3′) and RAD51-1L (5′- CTCCCCTTCCAGAGAAATCTG -3′) and mutant allele using RAD51-1U and LBgabi1 (5′-CCCATTTGGACGTGAATGTAGACAC-3′). We amplified *SDS* wild type allele using primers N806294U (5′-CTGCTCCCTGATTACAAGCAG-3′) and N806294L (5′-CTTAACGCATTCAGGCAACTC-3′) and mutant allele using N806294U and Lb3sail. *At*MSH4 wild type allele was amplified using primers N636296U (5′-CTTCTTGCAGGTTGTGTTTG-3′) and N636296L (5′-GCCAGCTGTTTTTGTTGTC-3′) and mutant allele using N636296L and LbSalk2. *At*MUS81A wild type allele was amplified for Salk_107515 using primers N607515U (5′-CATGCTGACAGTTGAAGGTC-3′) and N607515L (5′-CCTCAAACGTTTCTCCAAAT-3′) and mutant allele using N607515L and LbSalk2. *At*MUS81A wild type allele was amplified for Salk_002176 using primers N502176U (5′-CACATACGTTTTTGGTTCCC-3′) and N502176L (5′-AGTGTCCAAGTCCTGCTTTC-3′) and mutant allele using N607515L and LbSalk2. *At*ZIP4 wild type allele was amplified using primers N568052U (5′-TCCTTCCCACACCTTGACCC-3′) and N568052L (5′-GACTGCTGGAGCAGAAACT-3′) and mutant allele using N568052L and LbSalk2. ASY1 wild type allele was amplified using primers N546272U (5′-TCTATGTTTGTTACGCGTTAATCAG-3′) and N546272L (5′-AGGTGGCTCGTAATCTGGTGGCTGC-3′) and mutant allele using N546272L and LbSalk2. SHOC1 wild type allele was amplified using primers N557589U (5′-TTACCGGAGTTTGAAAACCG-3′) and N557589L (5′-GGCAAAGACTTGAAGGCATC-3′) and mutant allele using N557589L and LbSalk2. *At*RAD1 was amplified using primers o629 (5′-CTGGTGAAGAACATTTGGTAG-3′) and o630 (5′-CTCTTATGGCTGCTGCGTCTTC-3′). Polymorphism between wild type and mutant alleles was revealed with Dde1 digestion.

### Fluorescent tagged lines

FTL lines were obtained from G.P. Copenhaver. For this study, we used two couple of adjacent intervals: I5aI5b and I5cI5d [31]. The procedure to create plants of interest and to collect data was described in [31], [53]. Statical analysis was performed as described in [31].

### Cytology, immunolocalization, and antibodies

Alexander staining for pollen viability was performed as described [54]. The protocol described by [55] was used to observe the female meiosis and the protocol described by [29] for male meiotic spreads. Immunolocalization of *At*MLH1 was made as described by [29]. Immunolocalization of *At*ZYP1 and *At*DMC1 was performed according to [56] with the modifications described in [43]. The anti-ASY1 polyclonal [56] and anti-ZYP1 polyclonal [49] antibodies were used at a dilution of 1∶250. The anti-MLH1 antibody [29] was used at a dilution of 1∶200. The anti-DMC1 antibody was described in [43] and the purified serum was used at 1∶20.

### Microscopy

For male meiotic spreads, observations were made with a Leica DM RXA2 epifluorescence microscope using an oil PL APO 100X/1.40 objective (Leica). Photographs were taken using a CoolSNAP HQ (Roper Scientific) camera driven by Open-LAB 4.0.4 software (Improvision). For immunocytology and FTLs analyzes, observations were made using a Zeiss Axio Imager2 microscope. We analyzed FTLs using the automatic slide-scanner function of the ZEISS AxioObserver DIC FISH Apotome and its workbench. Photographs were taken using an AxioCam MRm (Zeiss) camera driven by Open-LAB 4.0.4 software AxioVision 4.8. All pictures were processed with AdobePhotoshop 7.0 (Adobe Systems Inc.).

## Supporting Information

Figure S1 A clustalW multiple alignment of the MCM8 protein family representatives. Black and grey shading indicate amino acid identical or similar, respectively (BLOSUM62) in at least 50% of the proteins. *At* (*Arabidopsis thaliana* MCM8), *Hs (Homo sapiens* MCM8 NP_115874.3), *Dm* (*Drosophila melanogaster* REC NP_732072.1), *Es* (*Entamoeba histolytica* MCM8 EAL48818.1), *Lm* (*Leishmania major* MCM8 CAB89596.2), *Pf* (*Plasmodium falciparum* MCM8 NP_701477.1).
(PDF)

Figure S2 Coimmunolocalization of ASY1 and *At*MLH1. ASY1 (red), *At*MLH1 (green) are shown as well as the overlay of both signals (merge) at diplotene in (A) wild type and in (B) *Atmcm8* mutant. Bar, 10 µm.
(TIF)

Figure S3 DMC1 immunolocalization. DNA (DAPI, blue) and *At*DMC1 (green) are shown as well as the overlay of both signals (merge) at zygotene in (A) wild type and in (B) *Atmcm8* mutant. Bar, 10 µm.
(TIF)

## References

[1] Cromie Ga, Smith GR (2007) Branching out: meiotic recombination and its regulation. Trends in cell biology 17: 448–455 doi:10.1016/j.tcb.2007.07.007. 17719784 10.1016/j.tcb.2007.07.007

[2] Keeney S (2008) Spo11 and the formation of DNA double-strand breaks in meiosis. Recombination and meiosis 81–123 doi:10.1007/7050. 10.1007/7050_2007_026 PMC3172816 21927624

[3] De Muyt A, Vezon D, Gendrot G, Gallois J-L, Stevens R, et al (2007) AtPRD1 is required for meiotic double strand break formation in Arabidopsis thaliana. The EMBO journal 26: 4126–4137 doi:10.1038/sj.emboj.7601815. 17762870 10.1038/sj.emboj.7601815 PMC2230667

[4] Kagawa W, Kurumizaka H (2010) From meiosis to postmeiotic events: uncovering the molecular roles of the meiosis-specific recombinase Dmc1. The FEBS journal 277: 590–598 doi:10.1111/j.1742-4658.2009.07503.x. 20015079 10.1111/j.1742-4658.2009.07503.x

[5] Cloud V, Chan Y-L, Grubb J, Budke B, Bishop DK (2012) Rad51 Is an Accessory Factor for Dmc1-Mediated Joint Molecule Formation During Meiosis. Science 337: 1222–1225 doi:10.1126/science.1219379. 22955832 10.1126/science.1219379 PMC4056682

[6] Li W, Chen C, Markmann-Mulisch U, Timofejeva L, Schmelzer E, et al (2004) The Arabidopsis AtRAD51 gene is dispensable for vegetative development but required for meiosis. Proceedings of the National Academy of Sciences of the United States of America 101: 10596–10601 doi:10.1073/pnas.0404110101. 15249667 10.1073/pnas.0404110101 PMC489980

[7] Vignard J, Siwiec T, Chelysheva L, Vrielynck N, Gonord F, et al (2007) The interplay of RecA-related proteins and the MND1-HOP2 complex during meiosis in Arabidopsis thaliana. PLoS Genet 3: e176 doi:10.1371/journal.pgen.0030176. 10.1371/journal.pgen.0030176 PMC2014788 17937504

[8] Couteau F, Belzile F, Horlow C, Grandjean O, Vezon D, et al (1999) Random chromosome segregation without meiotic arrest in both male and female meiocytes of a dmc1 mutant of Arabidopsis. The Plant cell 11: 1623–1634. 10488231 10.1105/tpc.11.9.1623 PMC144309

[9] Kurzbauer M-T, Uanschou C, Chen D, Schlögelhofer P (2012) The Recombinases DMC1 and RAD51 Are Functionally and Spatially Separated during Meiosis in Arabidopsis. The Plant cell: 1–14. doi:10.1105/tpc.112.098459. 10.1105/tpc.112.098459 PMC3442587 22589466

[10] Caryl APP, Armstrong SJ, Jones GH, Franklin FCH (2000) A homologue of the yeast HOP1 gene is inactivated in the Arabidopsis meiotic mutant asy1. Chromosoma 109: 62–71. 10855496 10.1007/s004120050413

[11] Azumi Y, Liu D, Zhao D, Li W, Wang G, et al (2002) Homolog interaction during meiotic prophase I in Arabidopsis requires the SOLO DANCERS gene encoding a novel cyclin-like protein. The EMBO journal 21: 3081–3095 doi:10.1093/emboj/cdf285. 12065421 10.1093/emboj/cdf285 PMC126045

[12] De Muyt A, Pereira L, Vezon D, Chelysheva L, Gendrot G, et al (2009) A high throughput genetic screen identifies new early meiotic recombination functions in Arabidopsis thaliana. PLoS Genet 5: e1000654 doi:10.1371/journal.pgen.1000654. 19763177 10.1371/journal.pgen.1000654 PMC2735182

[13] Sanchez-Moran E, Santos JL, Jones GH, Franklin FCH (2007) ASY1 mediates AtDMC1-dependent interhomolog recombination during meiosis in Arabidopsis. Genes & development 21: 2220–2233 doi:10.1101/gad.439007. 17785529 10.1101/gad.439007 PMC1950860

[14] Osman K, Higgins JD, Sanchez-Moran E, Armstrong SJ, Franklin FCH (2011) Pathways to meiotic recombination in Arabidopsis thaliana. The New phytologist 190: 523–544 doi:10.1111/j.1469-8137.2011.03665.x. 21366595 10.1111/j.1469-8137.2011.03665.x

[15] Harrison CJ, Alvey E, Henderson IR (2010) Meiosis in flowering plants and other green organisms. Journal of experimental botany 61: 2863–2875 doi:10.1093/jxb/erq191. 20576791 10.1093/jxb/erq191

[16] Lynn A, Soucek R, Börner GV (2007) ZMM proteins during meiosis: crossover artists at work. Chromosome Research 15: 591–605 doi:10.1007/s10577-007-1150-1. 17674148 10.1007/s10577-007-1150-1

[17] Mézard C, Vignard J, Drouaud J, Mercier R (2007) The road to crossovers: plants have their say. Trends in genetics: TIG 23: 91–99 doi:10.1016/j.tig.2006.12.007. 17208327 10.1016/j.tig.2006.12.007

[18] Maiorano D, Lutzmann M, Méchali M (2006) MCM proteins and DNA replication. Current opinion in cell biology 18: 130–136 doi:10.1016/j.ceb.2006.02.006. 16495042 10.1016/j.ceb.2006.02.006

[19] Liu Y, Richards Ta, Aves SJ (2009) Ancient diversification of eukaryotic MCM DNA replication proteins. BMC evolutionary biology 9: 60 doi:10.1186/1471-2148-9-60. 19292915 10.1186/1471-2148-9-60 PMC2667178

[20] Maiorano D, Cuvier O, Danis E, Méchali M (2005) MCM8 is an MCM2-7-related protein that functions as a DNA helicase during replication elongation and not initiation. Cell 120: 315–328 doi:10.1016/j.cell.2004.12.010. 15707891 10.1016/j.cell.2004.12.010

[21] Volkening M, Hoffmann I (2005) Involvement of Human MCM8 in Prereplication Complex Assembly by Recruiting hcdc6 to Chromatin Involvement of Human MCM8 in Prereplication Complex Assembly by Recruiting hcdc6 to Chromatin. 25 doi:10.1128/MCB.25.4.1560. 10.1128/MCB.25.4.1560-1568.2005 PMC548026 15684404

[22] Lutzmann M, Grey C, Traver S, Ganier O, Maya-Mendoza A, et al (2012) MCM8- and MCM9-Deficient Mice Reveal Gametogenesis Defects and Genome Instability Due to Impaired Homologous Recombination. Molecular cell 47: 523–534 doi:10.1016/j.molcel.2012.05.048. 22771120 10.1016/j.molcel.2012.05.048

[23] Nishimura K, Ishiai M, Horikawa K, Fukagawa T, Takata M, et al (2012) Mcm8 and Mcm9 Form a Complex that Functions in Homologous Recombination Repair Induced by DNA Interstrand Crosslinks. Molecular cell 47: 511–522 doi:10.1016/j.molcel.2012.05.047. 22771115 10.1016/j.molcel.2012.05.047

[24] Blanton HL, Radford SJ, McMahan S, Kearney HM, Ibrahim JG, et al (2005) REC, Drosophila MCM8, drives formation of meiotic crossovers. PLoS Genet 1: e40 doi:10.1371/journal.pgen.0010040. 16189551 10.1371/journal.pgen.0010040 PMC1231718

[25] Page SL, Hawley RS (2004) The genetics and molecular biology of the synaptonemal complex. Annual review of cell and developmental biology 20: 525–558 doi:10.1146/annurev.cellbio.19.111301.155141. 10.1146/annurev.cellbio.19.111301.155141 15473851

[26] Armstrong SJ, Caryl APP, Jones GH, Franklin FCH (2002) Asy1, a protein required for meiotic chromosome synapsis, localizes to axis-associated chromatin in Arabidopsis and Brassica. Journal of Cell Science 115: 3645–3655 doi:10.1242/jcs.00048. 12186950 10.1242/jcs.00048

[27] Higgins JD, Sanchez-Moran E, Armstrong SJ, Jones GH, Franklin FCH (2005) The Arabidopsis synaptonemal complex protein ZYP1 is required for chromosome synapsis and normal fidelity of crossing over. Genes & development 19: 2488–2500 doi:10.1101/gad.354705. 16230536 10.1101/gad.354705 PMC1257403

[28] Stacey NJ, Kuromori T, Azumi Y, Roberts G, Breuer C, et al (2006) Arabidopsis SPO11-2 functions with SPO11-1 in meiotic recombination. The Plant Journal 48: 206–216 doi:10.1111/j.1365-313X.2006.02867.x. 17018031 10.1111/j.1365-313X.2006.02867.x

[29] Chelysheva L, Grandont L, Vrielynck N, le Guin S, Mercier R, et al (2010) An easy protocol for studying chromatin and recombination protein dynamics during Arabidopsis thaliana meiosis: immunodetection of cohesins, histones and MLH1. Cytogenetic and genome research 129: 143–153 doi:10.1159/000314096. 20628250 10.1159/000314096

[30] Jackson N, Sanchez-Moran E, Buckling E, Armstrong SJ, Jones GH, et al (2006) Reduced meiotic crossovers and delayed prophase I progression in AtMLH3-deficient Arabidopsis. The EMBO journal 25: 1315–1323 doi:10.1038/sj.emboj.7600992. 16467846 10.1038/sj.emboj.7600992 PMC1422170

[31] Berchowitz LE, Copenhaver GP (2008) Fluorescent Arabidopsis tetrads: a visual assay for quickly developing large crossover and crossover interference data sets. Nature protocols 3: 41–50 doi:10.1038/nprot.2007.491. 18193020 10.1038/nprot.2007.491

[32] Sekelsky JJ, McKim KS, Chin GM, Hawley RS (1995) The Drosophila meiotic recombination gene mei-9 encodes a homologue of the yeast excision repair protein Rad1. Genetics 141: 619–627. 8647398 10.1093/genetics/141.2.619 PMC1206761

[33] Liu Z, Hossain GS, Islas-Osuna MA, Mitchell DL, Mount DW (2000) Repair of UV damage in plants by nucleotide excision repair: Arabidopsis UVH1 DNA repair gene is a homolog of Saccharomyces cerevisiae Rad1. The Plant journal: for cell and molecular biology 21: 519–528. 10758502 10.1046/j.1365-313x.2000.00707.x

[34] Gallego F, Fleck O, Li A, Wyrzykowska J, Tinland B (2000) AtRAD1, a plant homologue of human and yeast nucleotide excision repair endonucleases, is involved in dark repair of UV damages and recombination. The Plant journal: for cell and molecular biology 21: 507–518. 10758501 10.1046/j.1365-313x.2000.00694.x

[35] Dubest S, Gallego ME, White CI (2002) Role of the AtRad1p endonuclease in homologous recombination in plants. EMBO reports 3: 1049–1054 doi:10.1093/embo-reports/kvf211. 12393748 10.1093/embo-reports/kvf211 PMC1307604

[36] Ravi M, Chan SWL (2010) Haploid plants produced by centromere-mediated genome elimination. Nature 464: 615–618 doi:10.1038/nature08842. 20336146 10.1038/nature08842

[37] Sanchez-moran E, Santos JL, Jones GH, Franklin FCH (2007) ASY1 mediates AtDMC1-dependent interhomolog recombination during meiosis in Arabidopsis. Genes & Development 1: 2220–2233 doi:10.1101/gad.439007.2002. 10.1101/gad.439007 PMC1950860 17785529

[38] Sheridan S, Bishop DK (2006) Red-Hed regulation: recombinase Rad51, though capable of playing the leading role, may be relegated to supporting Dmc1 in budding yeast meiosis. Genes & development 20: 1685–1691 doi:10.1101/gad.1447606. 16818601 10.1101/gad.1447606

[39] Tsubouchi H, Roeder GS (2006) Budding yeast Hed1 down-regulates the mitotic recombination machinery when meiotic recombination is impaired. Genes & development 20: 1766–1775 doi:10.1101/gad.1422506. 16818607 10.1101/gad.1422506 PMC1522073

[40] Niu H, Wan L, Busygina V, Kwon Y, Allen Ja, et al (2009) Regulation of meiotic recombination via Mek1-mediated Rad54 phosphorylation. Molecular cell 36: 393–404 doi:10.1016/j.molcel.2009.09.029. 19917248 10.1016/j.molcel.2009.09.029 PMC2788773

[41] Shinohara A, Gasior S, Ogawa T, Kleckner N, Bishop DK (1997) Saccharomyces cerevisiae recA homologues RAD51 and DMC1 have both distinct and overlapping roles in meiotic recombination. Genes to cells: devoted to molecular & cellular mechanisms 2: 615–629. 9427283 10.1046/j.1365-2443.1997.1480347.x

[42] Niu H, Li X, Job E, Park C, Moazed D, et al (2007) Mek1 kinase is regulated to suppress double-strand break repair between sister chromatids during budding yeast meiosis. Molecular and cellular biology 27: 5456–5467 doi:10.1128/MCB.00416-07. 17526735 10.1128/MCB.00416-07 PMC1952091

[43] Chelysheva L, Gendrot G, Vezon D, Doutriaux M-P, Mercier R, et al (2007) Zip4/Spo22 is required for class I CO formation but not for synapsis completion in Arabidopsis thaliana. PLoS Genet 3: e83 doi:10.1371/journal.pgen.0030083. 17530928 10.1371/journal.pgen.0030083 PMC1877879

[44] Schwacha a, Kleckner N (1997) Interhomolog bias during meiotic recombination: meiotic functions promote a highly differentiated interhomolog-only pathway. Cell 90: 1123–1135. 9323140 10.1016/s0092-8674(00)80378-5

[45] Youds JL, Boulton SJ (2011) The choice in meiosis - defining the factors that influence crossover or non-crossover formation. Journal of cell science 124: 501–513 doi:10.1242/jcs.074427. 21282472 10.1242/jcs.074427

[46] Alonso JM, Stepanova AN, Leisse TJ, Kim CJ, Chen H, et al (2003) Genome-wide insertional mutagenesis of Arabidopsis thaliana. Science (New York, NY) 301: 653–657 doi:10.1126/science.1086391. 10.1126/science.1086391 12893945

[47] Hartung F, Wurz-Wildersinn R, Fuchs J, Schubert I, Suer S, et al (2007) The catalytically active tyrosine residues of both SPO11-1 and SPO11-2 are required for meiotic double-strand break induction in Arabidopsis. The Plant cell 19: 3090–3099 doi:10.1105/tpc.107.054817. 17965269 10.1105/tpc.107.054817 PMC2174718

[48] Pradillo M, López E, Linacero R, Romero C, Cuñado N, et al (2012) Together yes, but not coupled: new insights into the roles of RAD51 and DMC1 in plant meiotic recombination. The Plant journal: for cell and molecular biology 69: 921–933 doi:10.1111/j.1365-313X.2011.04845.x. 22066484 10.1111/j.1365-313X.2011.04845.x

[49] Higgins JD, Armstrong SJ, Franklin FCH, Jones GH (2004) The Arabidopsis MutS homolog AtMSH4 functions at an early step in recombination: evidence for two classes of recombination in Arabidopsis. Genes & Development: 2557–2570. doi:10.1101/gad.317504.eukaryote. 10.1101/gad.317504 PMC529542 15489296

[50] Higgins JD, Buckling EF, Franklin FCH, Jones GH (2008) Expression and functional analysis of AtMUS81 in Arabidopsis meiosis reveals a role in the second pathway of crossing-over. The Plant Journal 54: 152–162 doi:10.1111/j.1365-313X.2008.03403.x. 18182028 10.1111/j.1365-313X.2008.03403.x

[51] Berchowitz LE, Francis KE, Bey AL, Copenhaver GP (2007) The role of AtMUS81 in interference-insensitive crossovers in A. thaliana. PLoS Genet 3: e132 doi:10.1371/journal.pgen.0030132. 17696612 10.1371/journal.pgen.0030132 PMC1941751

[52] Marimuthu MPa, Jolivet S, Ravi M, Pereira L, Davda JN, et al (2011) Synthetic clonal reproduction through seeds. Science 331: 876 doi:10.1126/science.1199682. 21330535 10.1126/science.1199682

[53] Macaisne N, Vignard J, Mercier R (2011) SHOC1 and PTD form an XPF-ERCC1-like complex that is required for formation of class I crossovers. Journal of cell science 124: 2687–2691 doi:10.1242/jcs.088229. 21771883 10.1242/jcs.088229

[54] Alexander M (1969) Differential staining of aborted and nonaborted pollen. Biotechnic & Histochemistry 44: 117–122. 10.3109/10520296909063335 4181665

[55] Motamayor J, Vezon D, Bajon C (2000) Switch (swi1), an Arabidopsis thaliana mutant affected in the female meiotic switch. Sexual Plant Reproduction 12: 209–218.

[56] Armstrong SJ (2002) Asy1, a protein required for meiotic chromosome synapsis, localizes to axis-associated chromatin in Arabidopsis and Brassica. Journal of Cell Science 115: 3645–3655 doi:10.1242/jcs.00048. 12186950 10.1242/jcs.00048

[57] Malkova A, Swanson J, German M, McCusker JH, Housworth Ea, et al (2004) Gene conversion and crossing over along the 405-kb left arm of Saccharomyces cerevisiae chromosome VII. Genetics 168: 49–63 doi:10.1534/genetics.104.027961. 15454526 10.1534/genetics.104.027961 PMC1448106

