# Supplementary material for: MCM8 Is Required for a Pathway of Meiotic Double-Strand Break Repair Independent of DMC1 in Arabidopsis thaliana
Source: PLoS Genet. 2013 Jan 3;9(1):e1003165. doi: 10.1371/journal.pgen.1003165 (PMC3536722; doi:10.1371/journal.pgen.1003165)

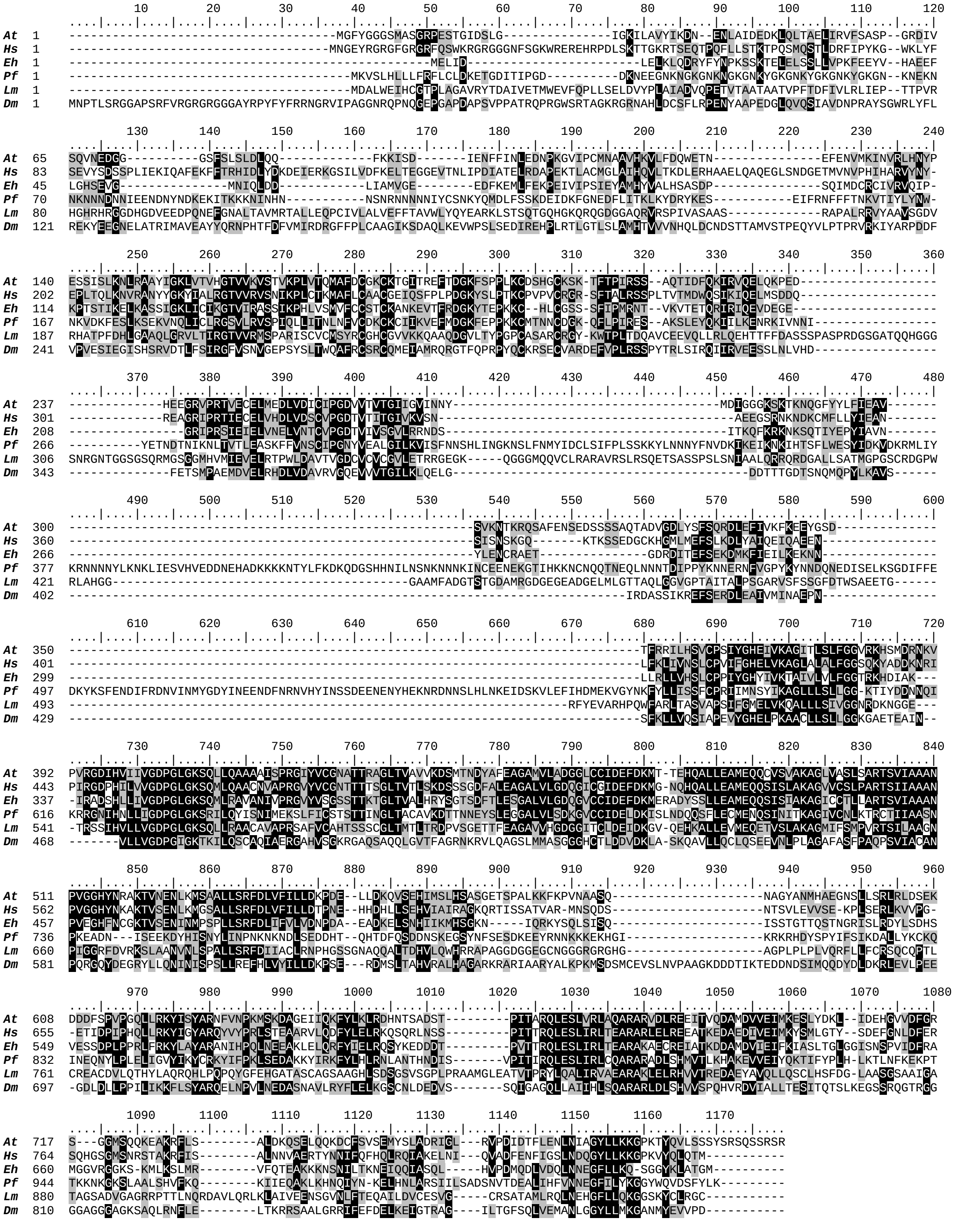

Supplement: Figure S1 — A clustalW multiple alignment of the MCM8 protein family representatives. Black and grey shading indicate amino acid identical or similar, respectively (BLOSUM62) in at least 50% of the proteins. At (Arabidopsis thaliana MCM8), Hs (Homo sapiens MCM8 NP_115874.3), Dm (Drosophila melanogaster REC NP_732072.1), Es (Entamoeba histolytica MCM8 EAL48818.1), Lm (Leishmania major MCM8 CAB89596.2), Pf (Plasmodium falciparum MCM8 NP_701477.1). (PDF) [file pgen.1003165.s001.pdf]
