# Supplementary figures and images for: MCM8 Is Required for a Pathway of Meiotic Double-Strand Break Repair Independent of DMC1 in Arabidopsis thaliana
Source: PLoS Genet. 2013 Jan 3;9(1):e1003165. doi: 10.1371/journal.pgen.1003165 (PMC3536722; doi:10.1371/journal.pgen.1003165)

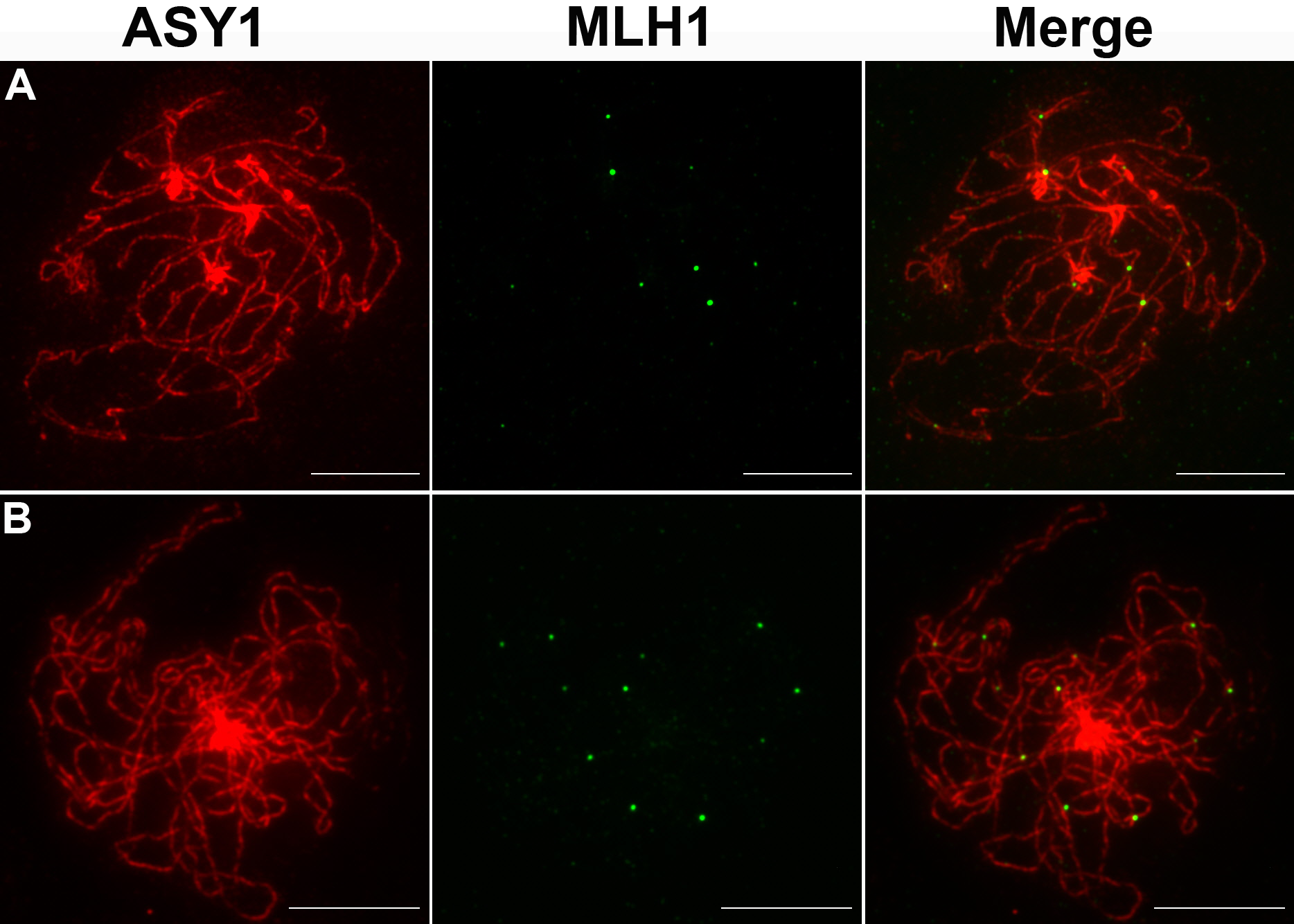

Supplement: Figure S2 — Coimmunolocalization of ASY1 and AtMLH1. ASY1 (red), AtMLH1 (green) are shown as well as the overlay of both signals (merge) at diplotene in (A) wild type and in (B) Atmcm8 mutant. Bar, 10 µm. (TIF) [file pgen.1003165.s002.tif]

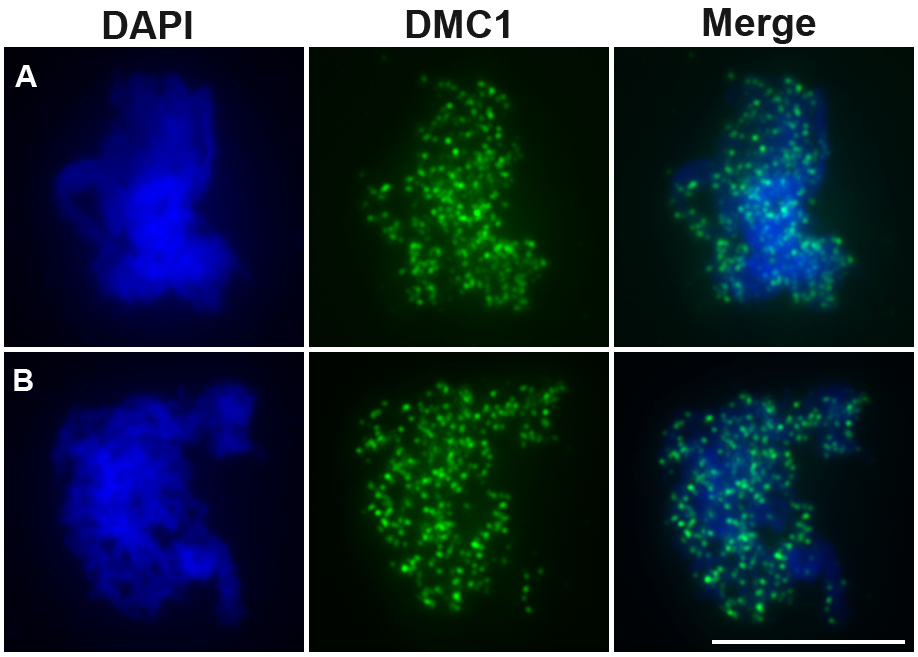

Supplement: Figure S3 — DMC1 immunolocalization. DNA (DAPI, blue) and AtDMC1 (green) are shown as well as the overlay of both signals (merge) at zygotene in (A) wild type and in (B) Atmcm8 mutant. Bar, 10 µm. (TIF) [file pgen.1003165.s003.tif]
